# The current state of welfare, housing and husbandry of the English pet rabbit population

**DOI:** 10.1186/1756-0500-7-942

**Published:** 2014-12-22

**Authors:** Nicola J Rooney, Emily J Blackwell, Siobhan M Mullan, Richard Saunders, Paula E Baker, Jenna M Hill, Clare E Sealey, Matthew J Turner, Suzanne DE Held

**Affiliations:** Department of Clinical Veterinary Science, Animal Welfare and Behaviour Group, University of Bristol, Langford, BS40 5DU Bristol, UK; CAPITA Secure Information Solutions, Methuen Park, Bath Rd, Chippenham, Wiltshire, SN14 0TW England

**Keywords:** Pet rabbit, Survey, Husbandry, Companionship, Health, Behaviour, Diet, Housing, Welfare

## Abstract

**Background:**

The welfare of pet rabbits is an area of growing interest in Europe and the UK. This study analyses questionnaire results from a diverse population of 1254 rabbit owners from three different geographical areas in England with the aim of providing an accurate representation of how pet rabbits are currently housed and cared for and key aspects of their health and welfare.

**Results:**

Rabbits were kept in a variety of different housing types, the most common being a traditional hutch/cage (59%). Although the majority had additional exercise areas, access was often unpredictable, or ill-timed, which may compromise welfare. Only 41.9% of owners kept their rabbit with conspecifics, limiting their ability to engage in social behaviour. Of those rabbits housed with a companion, although many were reported to be amicable and to engage in positive interactions, over a quarter were reported to fight at least occasionally (25.3%), whilst 22.7% guarded resources and 27.1% avoided one another. Whilst low levels of some of these behaviours may be a normal part of social interaction, the relatively high levels reported here suggest that not all cohabiting pairs of rabbits are compatible, which is potentially a significant welfare issue.

Although the vast majority of owners fed hay for over 10% this was less than daily. Pelleted foods were very popular (71.4% at least daily) compared to commercial muesli mixes (32.6%). As in previous studies, dental problems were commonly reported (12.2% of rabbits); however, so were eye problems (12.9%), digestive problems (11.5%) and parasites (11.3%). A large proportion of rabbits (58%) were thought to be fearful of loud noises, and 61% were not reported as calm when handled by their owner, which may be a significant concern for this species.

**Conclusion:**

This study has confirmed and expanded on previous findings: many pet rabbits were found to be in good health, had compatible companions and were provided with enriched living areas. However, it also found numerous welfare issues that affect large numbers of pet rabbits. We suggest further studies are required exploring the accuracy of owner reports (which possibly under-report many problems) and prioritising the issues raised here.

## Background

Rabbits are common pets in many western European countries [1]. They are the third most popular mammalian pet in the UK [2], with an estimated 1.7 million kept in 4% of UK households. However, given their popularity, surprisingly little is known about the way pet rabbits are kept and cared for [1], or how their housing and husbandry conditions affect their health, behaviour and general wellbeing. Three recent studies have suggested that traditional housing and husbandry practices may detrimentally affect rabbit welfare. Schepers et al. [1], in a survey of 912 owners and direct observations of their rabbits, identified several potential welfare concerns, including small hutches, solitary housing, poor socialisation, inadequate and inappropriate diets, and a lack of veterinary care. However, their study was conducted in the Netherlands and it is not known how similar their population was to the UK pet rabbit population.

UK studies have to date been less extensive. Edgar and Mullan [3] surveyed 52 owners at point of sale, whilst Mullan and Main [4, 5] took direct measurements on 102 rabbits in their homes in the South West of England. They found that 20% were kept in hutches smaller than 0.54 m^2^, the minimum cage area recommended for laboratory and farmed rabbits (up to 6kg: Home Office [6]); nearly 50% were housed alone, and the most common health problem was dental disease, of which the majority of owners were unaware. The studies by Mullan and Main likely targeted particularly keen rabbit owners by recruiting via advertisements in local shops and newspapers and it is not known how the relatively small populations studied reflect the wider English population. However, they do highlight several potential welfare issues.

In 2011, (and repeated annually) the PDSA (People’s Dispensary for Sick Animals e.g. [2, 7]) conducted a UK-wide internet survey reaching 1,132 rabbit owners and reporting welfare concerns relating to *each* of the five welfare needs [8]. While the sample size was larger than Mullan and Main [4, 5], the survey was distributed only to owners with time and skills to access the internet which represents a skewed demographic. Also since it included questions about three species (dogs, cats, rabbits), the amount of information regarding rabbits was limited.

The present study aimed to provide a representative point sample of the current state of care and welfare of English pet rabbits, against which subsequent surveys could be compared, and change assessed.

A questionnaire was devised consisting of over seventy questions and was presented in three different forms: a) written form, distributed via pet care outlets, veterinary surgeries and schools; b) verbally usually over the telephone, and c) via the internet, recruited in multiple ways. Diversity of recruitment methods aimed to overcome some of the sampling bias inherent in past surveys. A prize draw was used to incentivise owners to take part.

Owners were asked about the way they housed and cared for their rabbits, collecting information on many factors hypothesised to affect their welfare, including housing type and size, exercise and grazing provision, husbandry and cleaning regimes, diet, companionship and veterinary care (e.g. vaccination, health checks).

Increasingly, animal welfare is measured in terms of outcome- rather than input- based measures (e.g. [9]). Therefore, we collected data on a range of potential indicators of rabbit welfare status; including indicators of physical health and disease, and behavioural indicators of welfare; data on in-cage behaviour, general temperament, and responses to potential stressors including handling and common fear-provoking stimuli. We asked owners about the occurrence of specific behaviours including those which we later classified as “positive” and “negative”. Positive behaviours were classified as those whose expression is known to be important for physical health (e.g. rearing up for musculo-skeletal health), and those which are thought to indicate positive affective states such as locomotory play behaviour [10] (e.g. “binkying” (running and hopping or twisting in the air)). Negative behaviours were those whose expression can be symptomatic of an underlying welfare issue (e.g. chewing the cage, head swaying). We categorised chin rubbing (usually territory marking) [11] and throwing objects as neutral. Throwing could be considered as negative, but we think it ambiguous as it could also be motivated by frustration, investigation or play.

Past estimates of longevity have been based on the age of rabbits at the time of surveys and have raised concerns about potentially low life expectancy (e.g. 2.2 years in [1]). To avoid underestimating longevity here, we asked respondents about the age at death of their last rabbit. We present the data collated for 1254 rabbit owners responding to our survey. The aim is to provide a description of current housing and husbandry practices for English pets and to highlight potential welfare issues.

## Methods

### Questionnaire content

The questionnaire included 76 questions divided into eight sections:

- Section A Respondent - gender, age, details of any children, number of rabbits currently owned, main carer;
- Section B Rabbit - name, sex, neuter status, age and who it was obtained for;
- Section C Companionship - whether rabbit lives with another animal, length of cohabitation and frequency with which they show specific social behaviours;
- Section D Housing - size and details of main living space, any attached and separate runs; information on the length, height and also the area of the home enclosure. The diversity of accommodation in which rabbits are kept has made some past survey findings difficult to interpret. Therefore we categorised the main living space as the area to which the rabbit had permanent access, but we also asked questions about any "separate" and "attached" runs to which the rabbit had intermittent access, with and without the necessity to be moved.
- Section E Husbandry - exercise and cleaning routines, in summer and winter;
- Section F Diet - frequency of feeding 12 different foodstuffs, and whether rabbit exhibits selective feeding;
- Section G Health - whether ten specific symptoms of ill health had ever, occasionally, or often been observed; whether the rabbit suffered from 15 common veterinary complaints, currently or in the last year, and whether it required veterinary treatment. Estimates of current teeth and nail condition.
- Section H Behaviour - owner’s opinion of how the rabbit reacts to a number of situations, including handling by themselves, children and other adults, approaching their living space, and five potentially fear-provoking stimuli e.g. loud noises and open spaces, and similarly the rabbits’ most common activity.

To avoid bias created by owners of multiple rabbits choosing to give information about all or the healthiest or most interesting rabbit, respondents were instructed to answer only about the rabbit whose name came first alphabetically. They were then given the opportunity to provide extra details on the social groupings of all additional rabbits they owned, and the age at death of their most recently deceased rabbit.

Many of the questions included component parts, hence a maximum of 326 variables were collected from each respondent.

#### Questionnaire distribution

The same questionnaire was presented via three forms; a printed version, one delivered verbally during telephone interviews and an internet version. Each included the same questions presented in the same order, but the formatting was slightly modified for the internet software system available.

All three questionnaires were distributed in rural and urban locations within three target regions:

- South West – centring around Bristol and North Somerset;
- North West – centring around Manchester and the Wirral;
- Eastern – centring around Norwich and Eastern Norfolk.

### Written questionnaire

A total of 1419 written questionnaires, accompanied by prepaid return envelopes and advertising posters were placed in 82 different prominent locations, in veterinary surgeries, pet shops, RSPCA (Royal Society for the Prevention of Cruelty to Animals) clinics, rehoming centres, and other pet-related outlets. Regular checks were made at each outlet, to replenish supplies. Questionnaires were also given to rabbit-owning families in four schools, and to friends and family of the research team. In total, 343 owners responded, giving a response rate of 24.2%.

### Telephone/verbal questionnaire

Veterinary surgeries in each target area were contacted, and ten (including both surgeries located within pet stores and independent practices) agreed to contact their rabbit-owning clients to recruit survey respondents. In addition, sign-up lists were placed in three PDSA clinics and all rabbit-presenting clients were asked to participate. Recruitment lists were displayed at a very large rabbit show, postcards handed out at an education stall in Manchester and by RSPCA inspectors on routine calls and were sent to owners who had recently acquired a rabbit from a re-homing centre. In total, 484 owners volunteered. They were contacted by telephone at a convenient time, and 335 interviews taking between 5 and 80 minutes, were conducted (mean = 27.8 ± 9.1 mins).

### Internet questionnaire

The survey was linked to the University of Bristol homepage and publicised in each key region via radio, newspapers, fliers at agricultural shows, local shops, libraries and community centres, emails to staff of several companies and to members of the Rabbit Welfare Association and Fund; alerts on University of Bristol payslips, postings on Facebook and twitter pages, money-saving websites, and general pet and rabbit forums. A total of 1490 rabbit owners responded.

### Data handling

Responses from the internet questionnaire were downloaded and exported to Excel, telephone and written responses were entered into the same spreadsheet. All those respondents from outside the key areas were excluded. Descriptive analysis was carried out using SPSS v19.

Research was approved by the approved the University of Bristol ethics committee and was performed in accordance with the Declaration of Helsinki.

## Results

### The sample

Between January and July 2011, 2169 completed surveys were returned (Table 1), 915 were from outside the key areas so were excluded leaving a sample of 1254 owners (Table 2).

**Table 1:** **Composition of the complete sample of returned questionnaires – reported as number of respondents**

	Verbal	Written	Internet	TOTAL
South West	130	136	305	571
North West	103	129	157	389
Eastern	100	77	117	294
Other	2	2	911	915
Total	335	344	1490	2169

**Table 2:** **Recruitment sources of the1254 completed questionnaires from within the three key recruitment areas**

	Verbal	Written	Internet	Total
Veterinary surgeries	243	150	3	396
Facebook	0	0	159	159
Word of mouth	1	0	100	101
Newspapers	0	0	90	90
Pet shops	1	90	0	91
RSPCA or PDSA clinics	42	5	12	59
RSPCA mailing or website	0	0	45	45
Rabbit Welfare Association and Fund	0	0	44	44
Sign up sheet, show or vets	35	2	1	38
Postcards	9	0	23	32
Friends and family	2	22	5	29
University of Bristol website	0	0	25	25
Schools	0	21	0	21
Rabbit focussed website or forum (not RWAF)	0	0	21	21
Other animal centred outlet e.g. kennels	0	11	7	18
Other charities e.g. PDSA	0	12	1	13
University employee payslip	0	0	11	11
Flier picked up in shops etc.	0	1	7	7
Twitter	0	0	6	6
Agricultural show	0	0	4	4
Work email (not University)	0	0	4	4
Non- animal websites e.g. money saver	0	0	8	8
Radio	0	0	2	2
General pet websites	0	0	1	1
Unknown	0	28	0	28
**Total**	333	342	579	1254

### Respondents

Respondents were mainly female (89.1%) in the age range 30–49 (56.5%). At the time, 48.3% had no children in the household, whilst 45.6% had children permanently and 6.1% visited at least once every two weeks. More respondents had girls, either under (63.2%) or over ten (54.5%), than boys of the same ages (58.6 and 40.4%).

For those 877 owners who stated how many rabbits they owned, answers ranged from one to 37, two rabbits was most common (44.4%) and only 3.3% of owners owned more than six. Most were cared for by the person answering the questionnaire (53.1%), whilst 24.7% of respondents shared care with another adult and 16.4% with a child.

### Rabbits

In total, 58.5% of the rabbits were male, 40.9% female and 0.2% of unknown sex (0.4% of owners failed to answer). A reported 64.9% of males and 51% of females (overall 59.1% ) were neutered, for 0.5% the neuter status was unknown. Rabbits ranged in age from 2 months to 12. 7 years, (mean = 3.2 ± 2.3 years). The mean length of ownership was 2.9 (±2.2) years.

**Figure 1:**
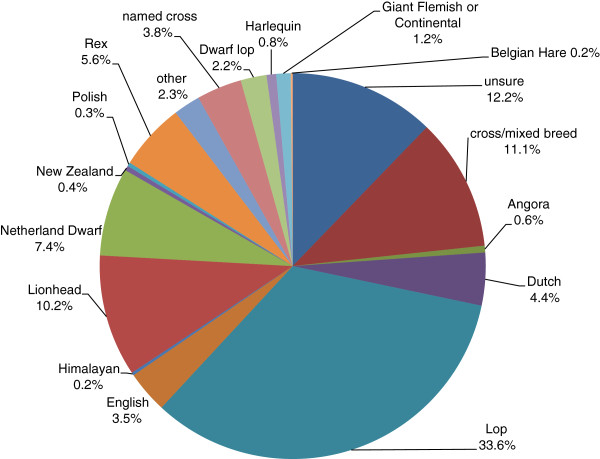
**Reported breeds/types of the 1254 rabbits in the questionnaire survey.**

The most common breed/type were Lops, whilst Lionheads, Netherland dwarfs and mixed breeds were also popular (Figure 1). A total of 28.4% of rabbits were described as dwarf, 51.5% as small, 17.3% as large and 2.6% as giant.

The most popular source of rabbits was a pet shop or garden centre (39.1%), followed by rehoming centre (17.6%), breeder (15.8%), friend or neighbour (15%), advert (4%), whilst 2.1% were found as strays. Most rabbits were obtained for the respondents (49%), but 27.6% were mainly for a child.

### Housing

**Figure 2:**
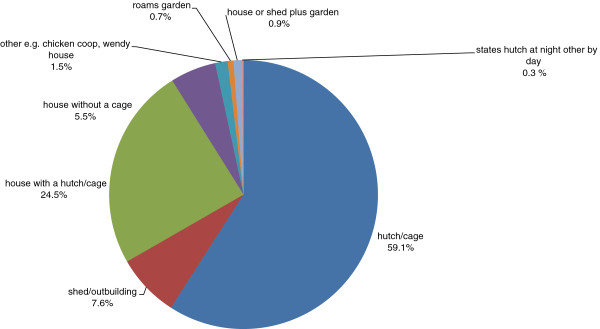
**Reported description of main living space.**

The majority of owners described their rabbit’s main living space as a hutch or cage (59.1%), whilst 29.9% were described as house rabbits, although most of these (82%) had a cage/hutch, but 5.5% of rabbits lived in the house, without any cage at all (Figure 2). A total of 59.5% of the rabbits lived mainly outdoors, 27.6% predominantly indoors and 12% in a shed, garage or outbuilding. For 19.5% location varied with season.

Most hutches/cages were on a single level (53.5%), 42.3% were over two and 4.2% had three or more levels. The surface area of the main hutch or cage, (including multiple levels if applicable, and the attached run or exercise area only if the rabbit had continual access to it), ranged from 0.1 m^2^ to 150503 m^2^, with a median size of 1.8 m^2^ (25th percentile = 0.9 m^2^, 75th percentile = 3.78 m^2^). When the number of animals in the enclosure was taken into consideration, the average space was 1.27 m^2^ per animal (0.72 m^2^, 2.57 m^2^), but 8.2% of the population (95 rabbits) were provided with less than 0.54 m^2^.

Six cages were recorded as over 10 m tall (presumably roofless). When these were removed the median height was 0.9 m (0.6, 1.2), but for 8.3% of the rabbits their main living space (with permanent run included only if they had constant access to it) was less than 0.5 m high throughout and for 42.1% it was less than 0.75 m. Within their home cage, 86.7% were reported to have a separate area for denning/sleeping, 71.1% had shelters, tunnels or boxes, 65.9% platform(s), and 59.2% had continual access to toys.

The vast majority of rabbits (97.5%) had some exercise area outside their home-cage, but the frequency and duration of access varied greatly. In total, 43.4% had an attached run (area they could reach without being moved by the owner); the average area being 198.59 (±406.3) m^2^. Of these 42.1% (23.5% of population) had continual access; 43.3% were shut away at night and for 8.8%, run use varied with the weather.

Separate runs were more popular; 62.9% of rabbits having access in summer, most commonly every day (25.2% of all rabbits), and for 4–8 hours (30.9%). However for 42.2% of rabbits, the frequency of access differed with the season; in winter only 49.9% had any access, with occasional (16.7%) 1–2 hour sessions being most frequently reported (11.1%). The mean run size was 30.56 (±90.63) m^2^, and whilst inside, 84.6% of rabbits had access to shelter, tunnel or box; 87.9% had grass to eat and 74% had ground to dig.

A reported 62.2% of rabbits were allowed to run loose in the garden, although only 50% in winter. For 22.8%, access was daily, or several times daily 8%; for the remainder, sessions were less predictable. Free running in the house was reported by 59% of owners.

### Companionship

Although 43.5% of rabbits were described as living alone, only 41.9% lived with other rabbit(s), whilst others lived with guinea pigs (4.3%), cats, dogs, quail, chickens, chameleons and even kune kune pigs. Rabbits living with conspecific companions were reported to show a variety of social behaviours (Table 3). Resting in contact, grooming and playing were very common, but considerable numbers of rabbits were also reported to mount, pull fur out and fight, at least on occasion.

**Table 3:** **Percentage of pair and group -housed rabbits reported to show social behaviours at differing frequencies**

	Never	Occasionally	Often
Playing with each other	5.3	31.8	62.9
Fighting (biting or scratching)	74.7	22.9	2.4
Resting in contact with each other	1.8	4.7	93.5
Grooming each other	4.4	12.1	83.5
Pulling out fur from each other	62.6	29.4	8.0
Mounting each other	34.4	51.6	14.0
Guarding items or areas from each other	77.3	19.0	3.7
Avoiding each other	72.9	25.8	1.3
Circling around each other	53.6	35.6	10.8
Chasing each other	22.5	54.8	22.7

### Husbandry routines

In the summer, most owners cleaned the cage weekly (44.6%) or more (27.2%), and took the soiled material out daily (50.2%) or more than once a week (27.2%). However 2.6% reported never thoroughly cleaning their rabbit’s living space. When cleaning, 45.2% sometimes (29.7%) or always (13.8%) left bedding inside, most used disinfectant occasionally (42.2%) or always (41.3%).

Most rabbits were handled at least weekly (Table 4), but less than half were handled daily. Many rabbits were groomed weekly, but a large proportion were never groomed (Table 4).

**Table 4:** **Percentage of respondents reporting handling (by various) people, health checking and grooming at differing frequencies**

	N	Never	Less than once a month	Approx monthly	Approx weekly	Most days	Daily
Picked up and handled by respondent	1246	2.2	6.7	5.6	18.0	21.3	46.3
Picked up and handled by other adults	1239	24.1	15.1	9.6	20.0	15.3	16.0
Picked up and handled by children under 10	1241	72.0	9.2	3.5	7.4	3.2	4.8
Picked up and handled by children 11-18	1241	65.7	7.2	4.8	8.5	5.4	8.5
Groomed	1243	23.9	15.2	17.5	26.6	93	7.6
Inspected to check the length of nails	1242	5.3	12.9	32.4	35.4	7.5	6.4
Inspected to check the length of teeth	1232	11.5	25.4	29.6	26.3	3.9	3.9

### Feeding

The most popular food types were root vegetables (e.g. carrots; 95.7) and hay (98.3%; Table 5). However, 10.6% of respondents reported feeding hay less than daily. A total of 74.1% of owners fed pelleted-foods at least daily compared to 32.5% feeding commercial muesli mixes. Of rabbits fed mixes, 52% were reported to leave specific parts (e.g. the pellets). Grass (freshly picked or growing) was fed at least weekly by 66.9% of owners. The most popular “other foodstuffs” were bread/crackers (10%) human breakfast cereals (3.8%), biscuits (2.3%) and herbs (3.2%).

**Table 5:** **Percentage of respondents reporting feeding each of thirteen foodstuffs at differing frequencies**

	***Percentage of those responding reporting to feed with each frequency***						
	Never	Occasionally	Once a week	More than once a week	Daily	Several times a day	Constant access
Commercial rabbit mix (cereal pieces of different shapes and colours)	60.0	5.7	0.5	1.3	23.4	1.8	7.3
Pellet feed (pieces all same shape and colour)	17.6	5.5	0.3	2.5	47.4	8.9	17.8
Green vegetables e.g. cabbage, broccoli	5.1	7.5	7.0	22.7	46.9	7.9	3.0
Salad e.g. lettuce	57.2	15.7	6.5	10.3	7.8	1.5	1.1
Garden/wild plants e.g. dandelions, brambles	13.0	28.7	7.9	20.4	22.1	1.9	6.0
Grass freshly picked or growing	14.8	20.4	7.0	16.4	29.2	2.1	10.1
Root vegetables e.g. carrots	4.3	13.5	9.0	31.0	36.6	3.4	2.2
Grass clippings	77.3	14.6	1.9	2.6	2.2	0.4	1.1
Hay/dried grass	1.7	2.1	2.3	4.5	23.5	1.3	64.6
Fruit e.g. apples, pears	17.9	34.7	11.6	20.2	13.3	1.0	1.2
Rabbit treats	23.6	41.8	9.9	11.4	11.7	0.9	0.6
Gnawing blocks	8.7	16.6	4.2	5.3	9.7	0.6	54.9
Other	80.0	7.3	2.7	3.4	5.2	1.0	0.5

### Health care

Nail inspections most commonly occurred weekly whilst teeth inspections were most commonly monthly (Table 4). In total, 70.8% of rabbits were vaccinated, but only 11.7% were insured.

#### Indicators of physical health

The majority of owners judged their rabbit’s nails and teeth, to currently be “fine” (nails: 87.1% teeth: 91.2%), a small proportion were unsure (2.9% and 0.4% respectively), and some admitted that they were possibly (8%, 2.7%) or definitely (1.1%, 0.7%) overgrown.

Of the presented list of clinical signs, indicative of underlying health issues, dirty bottoms were the most common; being reported as occurring at least occasionally by 30.1% owners (Table 6). Swollen body parts/lumps were the least common (3.5%). Caecotrophs (defined as smaller, sticky, often darker droppings- the caecal faeces usually re-ingested for nutritional purposes) were occasionally seen by 53.6% of owners and often by 9.4%.

**Table 6:** **Percentage of rabbits reported to show each of 10 ill health symptoms at differing frequencies**

	N	Never	Occasionally	Often
Runny eyes	1236	85.4	11.7	2.9
Runny nose	1235	92.4	6.9	0.7
Lack of appetite/not eating	1230	80.7	19.0	0.3
Wet fur around mouth and/or chest (drooling)	1231	95.9	3.9	0.2
Dirty bottom or droppings in fur	1241	69.9	25.7	4.4
Intense scratching	1233	94.7	5.0	0.2
Matted or soiled fur	1232	86.5	11.5	1.9
Swollen parts of body or lumps	1232	96.5	3.0	0.5
Limping/problems moving	1233	96.0	3.1	0.9
Hay or fur in droppings	1225	91.7	7.4	0.9

Of the range of presented veterinary conditions (Table 7), digestive problems were the most commonly occurring over the last year (6.6%) whilst dental disease, and being overweight were the most common currently (4.8% and 7.5% respectively). Flystrike (88.5%), ear problems (88%) and dental disease (88%) were most commonly referred to a vet, whilst only 52% of overweight and 50-1% of rabbits with sore hocks or inside legs were taken to a vet. When asked specifically about their rabbit’s body condition, 12.1% were described as overweight (0.2% very), 86.5% were thought to be the correct weight and only 1.5% underweight.

**Table 7:** **Percentage of rabbits reported to have experienced each of 15 veterinary conditions, over differing time periods**

	Over a year ago	In the past year	Currently	% of affected animals taken to vet
Ear problems	2.29	1.31	0.33	88
Eye problems	4.56	4.32	399	74
Digestive problems (e.g. gut blockage)	4.27	6.64	0.82	75
Overweight	3.20	2.87	7.46	52
Underweight	1.23	1.31	1.15	74
Dental/teeth problems	3.76	3.59	4.82	88
Respiratory/breathing problems	1.23	2.13	1.31	80
Skin problems	2.54	1.96	0.65	70
Parasites (e.g. mites /fleas)	5.98	4.10	1.23	77
Fly strike (i.e. maggots)	1.15	0.74	0.00	88
Fight wounds	2.95	1.97	0.41	64
Urine or kidney infections	1.23	1.15	0.33	73
Soreness on inside of legs	0.25	0.57	0.33	50
Sore hocks (reddened areas or bleeding of underside of heels or feet)	1.31	0.82	0.66	51
Neurological problems (e.g. head tilt)	0.58	0.25	0.74	80

### Behavioural indicators of welfare

**Figure 3:**
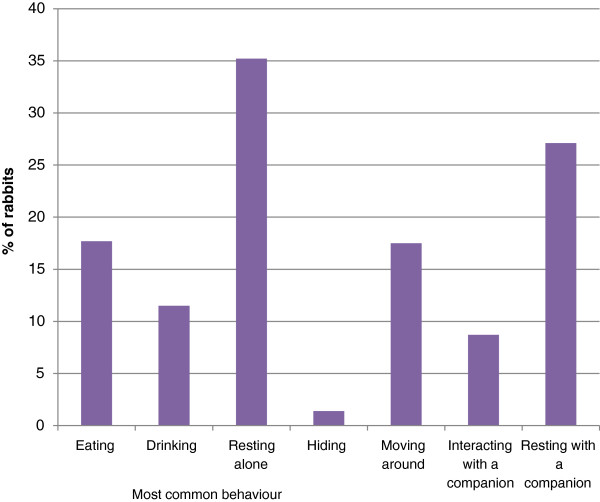
**Percentage of rabbits reported to spend the majority of time in each of seven activities.**

*In-cage behaviour* Most rabbits were described as spending most time resting alone (Figure 3; 35%) or resting with a companion (26.9%), moving and eating were relative common but a surprising 12% were believed to spend most time drinking.

**Figure 4:**
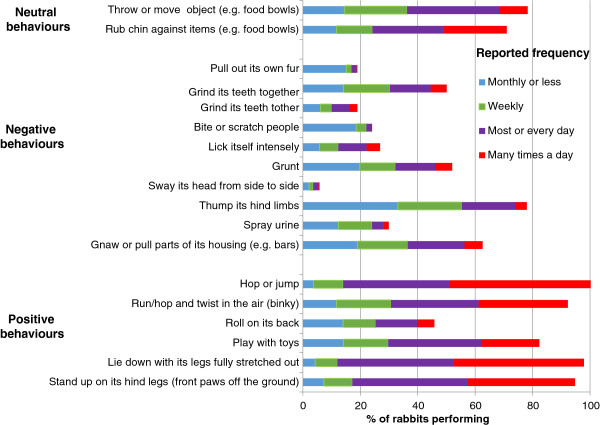
**Reported frequency of occurrence of a range of behaviours.**

Of the positive behaviours listed, most (Figure 4) (with the exception of rolling on their backs) were reported to be shown by the vast majority of rabbits, but at varying frequencies. However, the potentially negative behaviours of thumping hind limbs, grunting, guarding and digging on hard surfaces were also common.

### Human-directed behaviour

Most rabbits were thought likely to approach in a friendly manner (83.2%) when people approached their living area, but ignoring (25.2%), retreating (10.2%) or hiding (5.4%) were also common (some owners reported multiple responses). However, 1.7% of rabbits were thought likely to move towards the owner in an aggressive manner, and 2.6% to thump their hind legs or vocalise (2.2%).

When rating their own confidence at handling their rabbit 73% of owners scored 5 (very confident), 13% scored 4, 8% 3, 4% 2 and 2% scored 1, (not at all confident).

**Figure 5:**
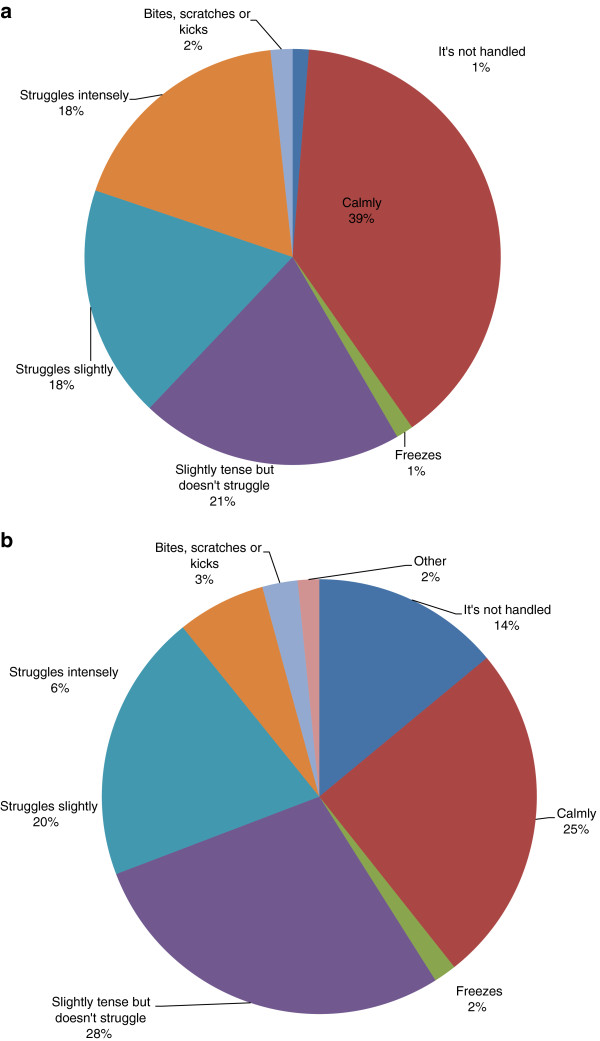
**Reported response to a) owner and b) other adults when handled.**

**Figure 6:**
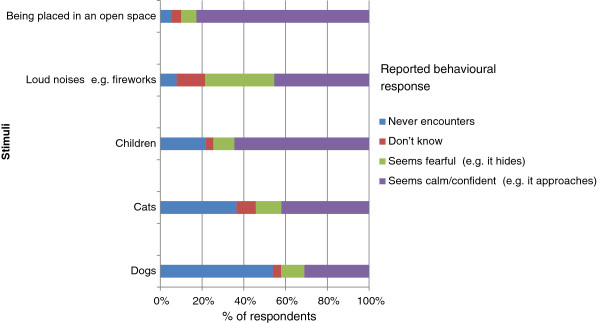
**Reported reactions to a range of potentially fear provoking stimuli.**

In total 39% of rabbits were described as calm when handled, but 61% of handled rabbits showed various signs of fear, (Figure 5); only 25% were described as calm when handled by other adults. The most commonly reported fear-provoking stimuli were loud noises (Figure 6).

### Longevity

Overall 830 owners provided data on the age at death of their last rabbit, which ranged from one month to 12 years with a mean of 5.6 (±0.1) years.

## Discussion

The survey shows the diversity in ways rabbits are kept and cared for and highlights a number of potential welfare issues in the English pet rabbit population. By recruiting in diverse ways and targeting less keen owners, we aimed to reach a more diverse sample of rabbit owners than have some past detailed surveys (e.g. [4]). Our sample is likely more representative of the general rabbit-owning population, although undoubtedly it is still skewed towards self-selected, keen rabbit owners and in particular by the large proportion of house rabbit owners (29%) who responded, and so the prevalence or severity of some problems may be underestimated.

Our survey shows that although the traditional hutch or cage remains the main living space of most English pet rabbits, rabbits are kept in diverse ways, including in Wendy houses, rooms and sheds. Patterns of access to living space also vary greatly between individuals. It is therefore important to understand how design and complexity, combined with size, affect welfare if guidance regarding optimal housing design is to be offered to rabbit owners, and this should be the focus of future research.

The results also show that although the majority of rabbits have additional exercise areas, less than a quarter of them are given continual access to these areas. We propose that the value of additional access areas varies with the timing and predictability of access, since unpredictable, uncontrollable routines have been shown to compromise welfare in numerous species (e.g. [12]). Patterns of access were often erratic (i.e. less than daily), and unpredictable, especially in winter, and environmental enrichment in this space is not universal. Since rabbits are naturally crepuscular [13], the timing of access to exercise areas (i.e. whether it occurs when the animal would naturally be most active) is likely to be integral to their welfare value. Many exercise areas contained no shelters or tunnels (15.4%). Since rabbits commonly show fear of open spaces (e.g. [14]), this may limit their value. Although the median cage size was considerably larger than minimum laboratory standards and similar to the PDSA survey [2]; and the number of rabbits living in small cages was less than that recorded by Mullan and Main [4], there were still nearly 10% with less than 0.54 m^2^ space available. It has been demonstrated that cages of 0.88 m^2^ limit the behaviours which rabbits can exhibit [15] and 27.5% of rabbits in this survey were kept in cages less than this area. Recommendations for pets include that cages should be sufficiently long to allow the animal to carry out three unrestricted hops, and to lie outstretched (e.g. [16]), many of the cages do not meet this suggestion.

Previous research has shown that rabbits sit and rear more in pens 75 cm high or with no ceilings, and the average height needed to fully ‘rear-up’ is 52.6 cm [15]. Platforms can encourage rearing and climbing and possibly improve musculoskeletal fitness, but 34.1% of rabbits were not provided with such devices. Large numbers of the surveyed rabbits would therefore be unable to rear regularly and consequent musculoskeletal health problems may arise. However rabbits also show behavioural preferences for enclosed areas [17], so the lack of tunnels (in 28.9% of cases) and denning areas (13.3%) in some enclosures is likely to also be a welfare concern.

Less than half of rabbits (42%) in this survey, and an even lower proportion of house-rabbits, lived with a conspecific. This figure is slightly higher than the recent PDSA [7] survey (35%), and highlights solitary housing as a potential welfare issue [1, 18, 19]. Rabbits are strongly motivated to gain social contact [20]. Solitary living precludes their ability to engage in normal social behaviour and negates one of the five basic needs, laid down in the Animal Welfare Act [8]. However, importantly, of those rabbits housed with a companion, over a quarter were reported to at least occasionally fight, whilst many guarded resources or avoided one another at least on occasion. Regular chasing and mounting were also common. Low levels of these behaviours may be a normal part of social interaction [21], but the relatively high levels reported here suggest that not all cohabiting pairs are compatible. Hence, to ensure welfare is improved, rather than compromised by living with a conspecific, it is essential that compatible pairings are selected and introduced appropriately, and adequately sized and structured living space is provided to allow rabbits to avoid one another if they so choose. The effects of mixing and incompatible rabbit combinations have been investigated within production systems (e.g. [22]), yet even the most comprehensive Code of Practice for pet rabbits [16] lacks a definition of “appropriate companion”, or details of the signs of compatibility as compared to incompatibility. Studies to determine the most compatible pet pairings, and optimal methods of introduction are required, as is advice to owners on how to monitor compatibility.

Our results show a marginal difference in food provision compared to past surveys as 40% of rabbits were reported to currently be fed commercial muesli mix, compared to 44% previously reported by Mullan and Main [4]. This could possibly indicate that past education campaigns warning of the potential links between muesli and obesity, dental disease and reduced water intake (e.g. [2, 16, 23]) may have had a small effect. However, over half of those feeding mix reported their rabbits selectively feeding on specific components, meaning they are unlikely to receive a balanced diet. For some rabbits, overfeeding of sugary foods such as root vegetables, treats and fruits (fed at least daily by 42.2, 13.2 and 15.5% of owners respectively) is a potential problem.

When grazing opportunities are limited, it is recommended that rabbit diets should consist primarily of high fibre forage, dried grass and hay. This is known to maintain intestinal physiology [24], to aid digestion, and to allow grazing behaviour which in wild counterparts occupies a large proportion of the day. Provision of forage is therefore believed to also prevent stereotypic behaviours [25, 26]. In total, 14.8% of rabbits were never fed grass, and 1.7% were never given hay or dried grass. This equates to 18 rabbits within this self-selected population, and a further 4.4% only received it weekly or less. Lack of, or inadequate (quantity or quality) of forage may be a contributory factor to a number of the veterinary and behavioural issues (e.g. dental disease; [27]; caecotroph appearance, obesity; [23]; repetitive behaviours) seen in this survey. Ongoing owner education about optimal rabbit feeding regimes is therefore still required.

Whilst this survey suggests that pet rabbits on average live longer than was previously reported (5.6 compared to 4.2 years in [1]), morbidity levels are high. Frequent symptoms of ill health were reported with perineal soiling (reported as “dirty bottoms”) being most common as has been previously suggested [28]. As seen in previous studies, dental problems were very common (12.2% reported to have suffered in this survey), but owners also described lack of appetite, runny eyes, digestive problems and many reported seeing caecotrophs, which may also be symptomatic of dental problems (or obesity), preventing the rabbits from consuming caecotrophs. The common occurrence of veterinary problems may, at least in part, be due to recruitment including distribution through veterinary surgeries. However dental disease was similarly found to be very common and under-reported by Mullan and Main [4] and our findings suggest that other issues may also be high and potentially unrecognised or underreported. Confirmation of causal factors, via additional research and improved education of carers regarding diet and health care is evidentially required.

Owners reported behaviours which were categorised as potentially indicative of “positive” welfare in the vast majority of rabbits, but with varying frequencies, for example although binkying was reported in 88%, only a quarter of rabbits were seen to do this many times per day. Behaviours like binkying, rearing up and lying stretched out may be prevented in some living environments (e.g. due to inadequate space or height) and they may also be under-reported in specific environments where owners are less likely to watch their rabbits, e.g. in runs compared to indoors. Potentially negative behaviours such as thumping hind limbs, gnawing housing, grunting and digging on hard surfaces were also common, which could be cause for concern. The relationship between the occurrence of these behavioural indicators and the rabbits’ environment and care warrant further research.

Within this sample, although most owners reported “picking up and handling” their rabbit at least weekly, a small minority of rabbits (2.2%) were never picked up and handled. It is however plausible that some of these were handled and stroked whilst on the floor, which can be less stressful to the rabbit and is generally good practice (e.g. http://www.bio.miami.edu/hare/firstrabbit.html). Hence we would recommend that future surveys make this distinction between “picking up” and “handling”.

Owner reports of behaviour suggest that most rabbits do not respond calmly when handled either by their owner (61%) or other adults (75%). Combined with the facts that 27% of owners do not describe themselves as “very confident” when handling their rabbit, yet the majority of rabbits are handled at least weekly; this could potentially represent a significant source of stress and suggests that appropriate handling protocols are essential to ensure this is not aversive, and that fearful animals do not become further sensitised to handling. The value of early positive handling has been demonstrated for laboratory rabbits (e.g. [29]) but further attention and research is needed to determine and promote optimal protocols for pets.

Over half of the pet rabbits were reported to be fearful of loud noises, suggesting that celebrations, for example involving fireworks, could be a significant welfare concern for this species, especially as they are more likely to be housed outdoors than other domestic pets. Research into ways to best habituate rabbits to and treat developed fears of loud noises would be very valuable.

Finally, it is worth noting that unlike in Mullan and Main [4], most rabbits in our survey had been obtained from pet shops or garden centres. Since Edgar and Mullan [3] noted that the conditions observed at the point of sale affected the way owners proposed to house their rabbits, it is likely that welfare education campaigns which engage pet shops and improve the way they exhibit rabbits will also positively affect how the public keep their pet rabbits.

## Conclusion

This large-scale survey gives us up-to-date in-depth information on the ways in which pet rabbits are housed and cared for in England.

Owner-reported data presented here suggest that whilst some rabbits have good health, compatible companions and are provided with enriched living areas, large numbers of rabbits experience potential stressors and welfare issues on a daily basis. The accuracy of the owners’ reports is yet to be tested, and it is likely that some of these issues are even more prevalent than this survey has suggested. Many rabbits are kept in ways which do not meet common recommendations (e.g. Wales [16]; Northern Ireland [30]).

This study has identified a large number of areas in which further research is required, for example prioritisation of the range of welfare issues highlighted by this survey, and to confirm postulated associations between aspects or housing and husbandry and rabbit welfare status. Future research also needs to focus on overcoming common issues, such as determining optimal protocols for habituating rabbits to human handling and to loud noises, which have been shown to be common causes of fear.
